# The Coupling of Peripheral Blood Pressure and Ventilatory Responses during Exercise in Young Adults with Cystic Fibrosis

**DOI:** 10.1371/journal.pone.0168490

**Published:** 2016-12-20

**Authors:** Erik H. Van Iterson, Courtney M. Wheatley, Sarah E. Baker, Thomas P. Olson, Wayne J. Morgan, Eric M. Snyder

**Affiliations:** 1 Department of Cardiovascular Diseases, Mayo Clinic, Rochester, MN, United States of America; 2 College of Pharmacy, University of Arizona, 1295 N Martin Ave, Tucson, AZ, United States of America; 3 Department of Anesthesiology, Mayo Clinic, Rochester, MN, United States of America; 4 Department of Pediatrics, University of Arizona, 1501 N. Campbell Avenue, Room 3301, Tucson, AZ, United States of America; 5 Department of Kinesiology, University of Minnesota, Cooke Hall, 1900 University Ave SE. Minneapolis, MN, United States of America; Universidad de Buenos Aires, ARGENTINA

## Abstract

**Purpose:**

Cystic fibrosis (CF) is commonly recognized as a pulmonary disease associated with reduced airway function. Another primary symptom of CF is low exercise capacity where ventilation and gas-exchange are exacerbated. However, an independent link between pathophysiology of the pulmonary system and abnormal ventilatory and gas-exchange responses during cardiopulmonary exercise testing (CPET) has not been established in CF. Complicating this understanding, accumulating evidence suggests CF demonstrate abnormal peripheral vascular function; although, the clinical implications are unclear. We hypothesized that compared to controls, relative to total work performed (Work_TOT_), CF would demonstrate increased ventilation accompanied by augmented systolic blood pressure (SBP) during CPET.

**Methods:**

16 CF and 23 controls (age: 23±4 vs. 27±4 years, *P* = 0.11; FEV_1_%predicted: 73±14 vs. 96±5, *P*<0.01) participated in CPET. Breath-by-breath oxygen uptake ($\dot{V}O_{2}$), ventilation (${\dot{V}}_{E}$), and carbon dioxide output ($\dot{V}CO_{2}$) were measured continuously during incremental 3-min stage step-wise cycle ergometry CPET. SBP was measured via manual sphygmomanometry. Linear regression was used to calculate ${\dot{V}}_{E}/\dot{V}CO_{2}$ slope from rest to peak-exercise.

**Results:**

Compared to controls, CF performed less Work_TOT_ during CPET (90±19 vs. 43±14 kJ, respectively, *P<*0.01). With Work_TOT_ as a covariate, peak ${\dot{V}}_{E}$ (62±8 vs. 90±4 L/min, *P* = 0.76), $\dot{V}CO_{2}$ (1.8±0.3 vs. 2.7±0.1 L/min, *P* = 0.40), and SBP (144±13 vs. 152±6 mmHg, *P* = 0.88) were similar between CF and controls, respectively; whereas CF demonstrated increased ${\dot{V}}_{E}/\dot{V}CO_{2}$ slope (38±4 vs. 28±2, *P* = 0.02) but lower peak $\dot{V}O_{2}$ versus controls (22±5 vs. 33±4 mL/kg/min, *P<*0.01). There were modest-to-moderate correlations between peak SBP with $\dot{V}O_{2}$ (*r* = 0.30), ${\dot{V}}_{E}$ (*r* = 0.70), and $\dot{V}CO_{2}$ (*r* = 0.62) in CF.

**Conclusions:**

These data suggest that relative to Work_TOT_, young adults with mild-to-moderate severity CF demonstrate augmented ${\dot{V}}_{E}/\dot{V}CO_{2}$ slope accompanied by increased SBP during CPET. Although the underlying mechanisms remain unclear, the coupling of ventilatory inefficiency with increased blood pressure suggest important contributions from peripheral pathophysiology to low exercise capacity in CF.

## Introduction

Mutation of the cystic fibrosis (CF) gene on chromosome 7 commonly leads to misfolding and/or improper transport of CF transmembrane conductance regulator (CFTR) within lung tissue at the epithelial cell level [1–4]. Because CFTR is important in maintaining transmembrane electrochemical gradient homeostasis, abnormal or absent CFTR is suggested to play a crucial role in the development of low airway function that is accompanied by reduced gas-transfer and abnormal ventilation, which are hallmarks of CF [1, 2, 5–7].

Key clinical correlates of CF morbidity and mortality are measurements of airway function including forced expiratory volume in one-second (FEV_1_) and forced vital capacity (FVC) [1, 2, 5–9]. While commonly measured at rest, a traditional paradigm in CF is that low FEV_1_ and FVC are suggested to underpin depressed oxygen uptake ($\dot{V}O_{2}$) commensurate with exacerbated ventilation during cardiopulmonary exercise testing (CPET) [9–14]. However, in contrast to this pulmonary-centric model of CF, though it is understood that intrinsic CF and/or non-specific factors complicate the pathophysiologic understanding of the adolescent versus adult CF clinical phenotype (e.g., age of first presentation of CF [15] versus intrinsic aging effects on cardiovascular function and/or CPET approach [16–18], respectively), noteworthy observations across the CF age and disease severity spectrum suggest these individuals demonstrate signs of attenuated exercise capacity hypothesized to be reflective of an appreciable contribution of non-pulmonary factors (both cardiac and peripheral) [8, 12–14, 18–25].

While the clinical translation to adult CF has yet to be elucidated, it is recognized in severe cardiopulmonary disease populations such as adult heart failure (HF) or chronic obstructive pulmonary disease (COPD) that indices of ventilatory efficiency representing multi-organ system function demonstrate prognostic strength consistent with, or surpassing peak $\dot{V}O_{2}$ ($\dot{V}O_{2peak}$) [26–28]. Specifically, reduced ventilatory efficiency described by a high slope of the ventilatory equivalent to carbon dioxide output ratio (${\dot{V}}_{E}/\dot{V}CO_{2}$ slope) during CPET strongly relates to HF or COPD patient clinical status and mortality risk [26–28]. From a physiologic perspective, ${\dot{V}}_{E}/\dot{V}CO_{2}$ slope has also been well-described across several lines of evidence as being reflective of integrated changes in cardiac, peripheral vascular, ventilatory, and gas-exchange function [26–29]. Moreover, in considering the practical implications of ${\dot{V}}_{E}/\dot{V}CO_{2}$ slope, it has been proposed this index is less influenced by non-physiologic factors during CPET such as participant effort, which is known to confound the interpretation of true $\dot{V}O_{2peak}$ in various patient or adolescent populations [28, 30, 31]. Lastly, by accounting for changes in systolic blood pressure (SBP) coupled with ${\dot{V}}_{E}/\dot{V}CO_{2}$ slope (termed ventilatory power, VPower), the long-term prognostic value of ${\dot{V}}_{E}/\dot{V}CO_{2}$ slope may be strengthened in patients with cardiopulmonary disease [29].

Although observations in adolescent or adult CF suggest in addition to impaired cardiac function [14, 22, 25, 32–34], these individuals may demonstrate abnormal peripheral function including skeletal muscle weakness or slowed muscle oxygenation kinetics [12, 18, 22, 23], which may cumulatively contribute to impaired aerobic exercise capacity coupled with ventilatory inefficiency, limited studies in adult CF have focused on describing the role of peripheral blood pressure [20, 35] in the context of questioning whether pulmonary dysfunction is accompanied by gross peripheral vascular abnormalities in these individuals [19, 21, 24, 36]. For example, whereas observations of Hull et al. [20] (ergometry) or Schrage et al. [35] (handgrip) do not suggest overt exacerbation of blood pressure during exercise in mild- to- moderate severity adult CF, it is both novel and relevant in understanding peripheral hemodynamic function in CF that Hull et al. [20] demonstrated increased augmentation index in these individuals, suggesting the presence of increased arterial stiffness. Further, and consistent with Hull et al. [20, 36], our group has demonstrated that mild- to- moderate severity adult CF have blunted reductions in resting systemic vascular resistance in response to inhalation of the β_2_-selective agonist albuterol [19]. Thus, if increased blood pressure, underpinned by impaired peripheral vascular function [19–21, 24, 35, 36], does indeed parallel exaggerated ${\dot{V}}_{E}/\dot{V}CO_{2}$ slope during exercise in adult CF, demonstrating this calibration in the laboratory setting should help add to our understanding that the clinical implications of CF may extend to the entire cardiovascular system.

This study aimed to test the hypothesis that relative to total work performed (Work_TOT_), compared to healthy adults, young adults with mild- to- moderate severity CF demonstrate an exaggerated ${\dot{V}}_{E}/\dot{V}CO_{2}$ slope (low ventilatory efficiency) that is coupled to direct relationships between increased SBP and ${\dot{V}}_{E}$ during CPET.

## Materials and Methods

### Participants

A sample of 16 adults with mild- to- moderate CF, confirmed by a positive sweat test (≥60 mmol/L sweat chloride) and genotyping of at least one ΔF508 CFTR mutation participated in this study (participant characteristics, Table 1). Individuals with CF were recruited through provider referrals from a CF clinic affiliated with the institutional medical center. Additionally, similar to others studies in adult CF [12, 14, 33, 34], a convenience sample of healthy adults (N = 23) were tested as controls who were recruited through word of mouth and posted advertisements around the institutional campus. No individual was involved in intense physical training or restrictive diet regimen prior to study participation.

**Table 1:** Participant characteristics.

	Controls	CF	*P*-value	ES
N	23	16	0.26	
Sex, male/female	12/11	12/4	0.15	
ΔF508 homozygous, N	-	12	-	
Age, years	27±4	23±4	0.11	0.55
Height, cm	173±5	169±4	0.18	0.46
Weight, kg	71±6	65±9	0.25	0.38
Body mass index, kg/m^2^	24±2	23±2	0.48	0.23
Body surface area, m^2^	1.8±0.1	1.7±0.1	0.16	0.46
Hemoglobin, g/dL	14.6±0.6	14.7±0.7	0.84	0.07
Serum Na^+^, mEq/L	139±1	137±1	0.01	0.91
Serum Cl^-^, mEq/L	104±1	103±2	0.18	0.45
Serum creatinine, mg/dL	1.01±0.08	0.93±0.09	0.26	0.50
eGFR, mL/min/1.73 m^2^	93±8	105±14	0.15	0.44
**Airway test parameters**				
Forced vital capacity, L	4.7±0.5	3.9±0.6	0.02	0.72
% predicted	97±4	83±10	0.01	0.80
FEV_1_, L	3.9±0.4	2.8±0.6	<0.01	1.02
% predicted	96±5	73±14	<0.01	1.08
FEV_1_/FVC	0.8±0.0	0.7±0.0	<0.01	1.02
FEF_25-75_, L/sec	3.9±0.4	2.3±0.8	<0.01	1.21
% predicted	94±8	57±18	<0.01	1.24
**Lung volume parameters**				
Total lung capacity, L	6.6±0.7	5.7±0.7	0.03	0.60
% predicted	105±6	92±6	<0.01	0.20
Vital capacity, L	4.9±0.6	4.0±0.7	0.03	0.67
% predicted	92±6	68±8	<0.01	0.53
Functional residual capacity, L	3.7±0.4	3.1±0.4	0.02	0.70
% predicted	105±8	89±7	<0.01	0.10
Residual volume, L	1.7±0.2	1.7±0.3	0.91	0.03
% predicted	119±22	114±18	0.77	0.64

Parametric raw data are mean ± 95% CL. Non-parametric raw data are N. CF, cystic fibrosis; ES, effect size; FEV_1_, forced expiratory volume in one second; FEF_25-75_ = forced expiratory flow at 25–75% of forced vital capacity. Parametric raw data compared using Wilcoxon rank-sum tests. Non-parametric raw data compared using χ^2^ tests.

To be eligible for participation in this study, all individuals with CF were required to have been diagnosed with CF, receiving adult-oriented care for their CF, and be clinically stable whereby individuals were excluded based on the following criteria: demonstrated a FEV_1_ ≤40 percent of predicted, experienced a pulmonary exacerbation within the last two weeks or pulmonary hemorrhage within six months resulting in greater than 50 cc of blood in the sputum, were taking any antibiotics for pulmonary exacerbation, currently receiving oral steroids, severe weight loss within the past 3 months (e.g., resulting in a body mass index <18 kg/m^2^) [37], or if they were taking any experimental drugs related to CF.

Additional exclusion criteria for all individuals prior to participation included: 1) medicated for the treatment of hypertension, cardiac, metabolic, diabetic, or neurologic diseases, 2) smoking history, 3) dependence on alcohol or recreational drugs, 4) being obese (i.e., body mass index >30 kg/m^2^ [38], 5) being pregnant, or 6) inability to engage in exercise. All aspects of this protocol were reviewed and approved by the University of Arizona Institutional Review Board. All individuals provided written informed consent prior to study participation.

### Protocol

Participants were asked to refrain from participating in exercise 24 hours prior to the study visit, consuming a meal 3 hours prior to the study visit, and consuming caffeine 8 hours prior to the study visit. All test measurements were performed in the upright position on a stationary upright cycle ergometer (Corival Lode B.V., Netherlands) on a single testing day in an environmentally controlled physiological laboratory.

Prior to CPET, resting airway function and lung volume testing via flow-volume loop spirometry were performed according to American Thoracic Society (ATS) guidelines [39, 40]. According ATS guidelines [41], ranges of both age and body anthropometry characteristics across participants suggested that calculation of percent of predicted airway function could be computed using standards from Hankinson et al. [42] or Crapo et al. [43]; whereas percent of predicted $\dot{V}O_{2peak}$ was calculated from equations of Hansen et al. [44], and percent of predicted peak heart rate (HR) from Tanaka et al. [45].

Upright CPET to volitional fatigue, which included continuous breath-by-breath ventilation and gas-exchange monitoring (MedGraphics CPX/D, Medical Graphics Corp, St Paul, MN), consisted of individualized incremental 3 min stages (workload increase ranged from 15 to 40 W, mean workload stage was 24±3 vs. 33±3 W in CF vs. controls, respectively; *P<*0.01) [46, 47]. The breath-by-breath system used for all CPET was calibrated according to manufacturer guidelines in the set-up used for testing prior to each participant test. This included calibration of the pneumotachograph through which participants inspired and expired through for linear flow across a range of flows, in addition to calibration of O_2_ and CO_2_ using medical grade gases of known concentrations. Pedal rate throughout CPET until volitional fatigue was 60 to 70 rpm. Rhythm and HR were continuously monitored using 12-lead electrocardiography (Marquette Electronics, Milwaukee, WI). Peripheral oxygen saturation was continuously measured via finger pulse oximetry (SpO_2_) (Nellcor N-600 Pulse Oximeter, Bolder, CO). According to the American College of Sports Medicine guidelines [31], measurement of both SBP and DBP by an experienced and certified exercise specialist occurred using manual sphygmomanometry at rest and during the final 30 seconds of each CPET stage. For analyses, basic ventilatory and gas-exchange indices were calculated as 30 second averages at rest, anaerobic threshold (AT), and for the final 30 seconds of CPET (i.e., peak exercise). Rate of perceived exertion (RPE, Borg scale, 6 to 20) was assessed at rest and at the end of each stage during CPET [48]. While criteria are not clearly defined in adult CF, which is inherent to the current study questions, despite several noteworthy approaches to determine $\dot{V}O_{2peak}$ in adolescent CF [47, 49–51], based on recommended guidelines from ATS and the American Heart Association for patients with impaired cardiopulmonary function, volitional fatigue consistent with achieving a $\dot{V}O_{2peak}$ was determined by an inability to maintain a constant pedal cadence between 60 to 70 rpm in addition to demonstrating a RPE >17, percent predicted HR >90%, or respiratory exchange ratio (RER) ≥1.10 [46, 52].

Derived variables included, mean arterial pressure (MAP) *=* (DBP + 1/3 (SBP−DBP)) and pulse pressure (PulseP *=* SBP−DBP). The slope of ${\dot{V}}_{E}/\dot{V}CO_{2}$ was computed separately from rest to AT in addition to peak exercise using all exercise data via linear regression as recommended by Arena et al. [26]. Anaerobic threshold was determined non-invasively using the V-slope technique developed by Beaver et al. [53]. Ventilatory power was calculated as the quotient of SBP and ${\dot{V}}_{E}/\dot{V}CO_{2}$ slope [29]. As an additional index of gross cardiovascular function, we calculated the quotient of $\dot{V}O_{2}$ and SBP ($\dot{V}O_{2}Power$) to describe the magnitude of change in $\dot{V}O_{2}$ relative to the change in SBP [54]. Ventilatory reserve (${\dot{V}}_{Ereserve}$) was calculated as the quotient of peak ${\dot{V}}_{E}$ and maximum voluntary ventilation (MVV = rest FEV_1_ ∙ 35) as a percentage [46]. Lastly, we quantified Work_TOT_ in kilojoules by calculating the area under the curve for W across CPET.

Invasive measurements of blood gases or hemodynamics were not collected during exercise to derive standard alveolar air equation parameters or assess ventilation and perfusion matching. However, it has been suggested by Hansen et al. that mixed expired CO_2_ (PECO_2_ = 863/[${\dot{V}}_{E}/\dot{V}CO_{2}$]) may be used to estimate the magnitude of ventilation and perfusion mismatch related to increased uneven ventilation and elevated physiologic deadspace to V_T_ ratios [55]. Thus, as a quotient with end-tidal partial pressure of CO_2_ (P_ET_CO_2_), low PECO_2_/P_ET_CO_2_ ratios (≤0.60) in the setting of severe airway disease is suggested to reflect low ventilation and perfusion ratios as well as high physiologic deadspace to V_T_ ratios [55].

### Statistical Analyses

Data met assumptions of homogeneous variance as tested using Levene’s test. Parametric raw data are presented as means ± 95% confidence limits [56]. Non-parametric raw data are presented as n. Wilcoxon rank-sum tests were used to compare participant characteristics between groups, except categorical variables (χ^2^-test). Between and within group differences for indices of interest were compared using two-factor ANCOVA with repeated measures models including rest, AT, and peak exercise data. Models included fixed group-by-time interactions. A continuous fixed effect of Work_TOT_ was set as a covariate in relevant models. In the event of significant F-tests, post-hoc calculations using Tukey-Kramer tests (appropriate for comparisons of unbalanced group sizes) were used to assess pairwise differences. Additional between group pairwise comparisons were performed using effect sizes (ES, Cohen’s *d*) according to methods of Cohen [57] and were interpreted as: 0.0 = trivial; 0.2 = small; 0.6 = moderate; 1.0 = large; and ≥2.0 = very large [57]. Pearson’s product moment correlation coefficient models were used to assess relationships (correlation coefficient, *r*) between peak blood pressure and peak measures of ventilation or gas-exchange. Standard interpretation of *r* from correlation models were based on thresholds of Cohen [57]: modest *r* = 0.10, moderate *r* = 0.30, and strong *r* ≥ 0.50. Two-tailed significance was determined using an alpha set at 0.05. Computations were made using SAS statistical software, version 9.4 (SAS Institute Inc., Cary, North Carolina).

## Results

Sixteen adults with mild- to- moderate severity CF as well as 23 healthy adults completed this study in the absence of adverse events (characteristics summarized, Table 1). Seventy-five percent of individuals with CF were homozygous for the ΔF508 CFTR gene mutation, whereas the remaining four individuals were heterozygotes for the ΔF508 CFTR gene mutation. Neither CF nor controls used bronchodilators (e.g., albuterol) <8 hours prior to airway testing, lung volume testing, or CPET. Individuals with CF were on standard pharmacologic therapy as recommended for CF [19]. No participant required use of therapy during testing procedures.

Although adult controls were recruited as part of a convenience sample and not selected based on specific body anthropometry except for the absence of obesity, in comparison to controls, CF demonstrated similar body anthropometry as well as blood biochemistry except for lower serum sodium levels (Table 1). In contrast, CF consistently demonstrated worse resting airway function and lung volumes compared to controls, highlighted by large- to- very large ES for FVC, FEV_1_, FEV_1_/FVC, and FEF_25-75_ (Table 1).

### Cardiopulmonary exercise testing

#### Exercise capacity

Metrics of exercise capacity including total exercise duration (933 ± 79 vs. 737 ± 113 s, *P<*0.01), peak workload (175 ± 22 vs. 109 ± 19 W, *P<*0.01), and Work_TOT_, were higher in controls compared to CF, respectively, despite similar RPE at peak exercise (Table 2). Similarly, $\dot{V}O_{2peak}$ (33 ± 4 vs. 22 ± 5 mL/kg/min, respectively, *P<*0.01; ES = 1.12), $\dot{V}O_{2peak}$ (both absolute and indexed to body surface area, Table 2), and percent predicted $\dot{V}O_{2peak}$ (97 ± 10 vs 58 ± 10%, respectively, *P<*0.01; ES = 1.72) were higher in controls compared to CF.

**Table 2:** Rest, anaerobic threshold, and peak exercise subjective and objective responses in controls and Cystic Fibrosis.

	Rest			Anaerobic Threshold			Peak Exercise		
	Controls	CF	ES	Controls	CF	ES	Controls	CF	ES
Total work performed, kJ	-	-	-	32±10	10±4	1.12	90±19^#^	43±14*^#^	1.27
RPE, Borg 6 to 20	6±0	6±0	0.00	13±1^‡^	12±1^‡^	0.42	18±0^†^	17±0^†^	0.42
SpO_2_, %	98±1	96±1	1.00	98±1	95±2	0.87	98±1	95±2	0.79
**Ventilation and gas-exchange**									
$\dot{V}O_{2}$, L/min	0.4±0.0	0.4±0.0	0.12	1.9±0.3^‡^	1.1±0.2*^‡^	1.47	2.5±0.3^†^^#^	1.5±0.3*^†^	1.40
$\dot{V}O_{2}$, L/min/m^2^	0.4±0.1	0.4±0.1	0.08	1.0±0.1^‡^	0.6±0.1*^‡^	1.37	2.3±0.3^†^^#^	1.4±0.3*^†^	1.39
$\dot{V}CO_{2}$, L/min	0.3±0.0	0.4±0.0	0.32	1.9±0.3^‡^	1.2±0.2*^‡^	1.46	2.7±0.1^†^	1.8±0.3^†^	1.40
$\dot{V}CO_{2}$, mL/kg/min	4.8±0.5	5.7±0.8	1.06	28±4^‡^	18±4^‡^	1.15	37±5^†^	25±5^†^	1.14
RER	0.81±0.06	0.91±0.08	0.73	1.02±0.02^‡^	1.01±0.03^‡^	0.19	1.12±0.03^†^^#^	1.11±0.03^†^^#^	0.16
${\dot{V}}_{E}$, L/min	15±2	17±2	0.44	57±7^‡^	43±4^‡^	1.13	90±4^†^	62±8^†^	1.34
${\dot{V}}_{E}$, mL/kg/min	209±35	265±47	0.67	811±97^‡^	671±80^‡^	0.74	1128±127^†^	891±112^†^	0.93
${\dot{V}}_{Ereserve}$, %	11±2	20±5*	1.08	43±4^‡^	53±16^‡^	0.46	59±5^†^	65±14^†^	0.32
RR, breaths/min	20±3	22±3	0.30	31±3^‡^	31±4^‡^	0.09	42±2^†^	40±9^†^	0.27
V_T_, L	0.8±0.1	0.8±0.2	0.00	1.9±0.2^‡^	1.5±0.2^‡^	0.91	2.2±0.1^†^	1.7±0.3^†^	0.83
V_T_, mL/kg	12±2	13±2	0.29	27±3^‡^	22±2^‡^^‡^	0.77	30±3^†^	26±3^†^	0.65
P_ET_CO_2_, mm Hg	31±1	30±1	0.12	37±2^‡^	34±2	0.50	35±2^#^	35±2	0.07
PECO_2_, mm Hg	21±1	19±1	0.29	29±2^‡^	23±3	1.09	27±1	25±2	0.69
PECO_2_/P_ET_CO_2_	0.7±0.0	0.7±0.0	0.76	0.8±0.0^‡^	0.7±0.0	1.00	0.8±0.0	0.7±0.0	1.00

Raw data are presented as mean ± 95% CL. Controls (n = 23); CF, cystic fibrosis (n = 16); ES, effect size; RPE, rate of perceived exertion; SpO_2_, oxygen saturation; $\dot{V}O_{2}$, oxygen uptake; $\dot{V}CO_{2}$, carbon dioxide output; ${\dot{V}}_{E}$, minute ventilation; ${\dot{V}}_{Ereserve}$, ventilatory reserve; RR, respiratory rate; V_T_, tidal volume; P_ET_CO_2_, end-tidal partial-pressure carbon dioxide; PECO_2_, mixed expired carbon dioxide.

* *P<*0.05, controls vs. CF.

^‡ ^*P<*0.05, rest vs. AT within group.

^† ^*P<*0.05, rest vs. peak exercise within group.

^# ^*P<*0.05, AT vs. peak exercise within group.

Significance after Tukey-Kramer post-hoc testing where appropriate.

#### ${\dot{V}}_{E}/\dot{V}CO_{2}$ slope, Ventilatory power, or Peak oxygen uptake power

**Fig 1:**
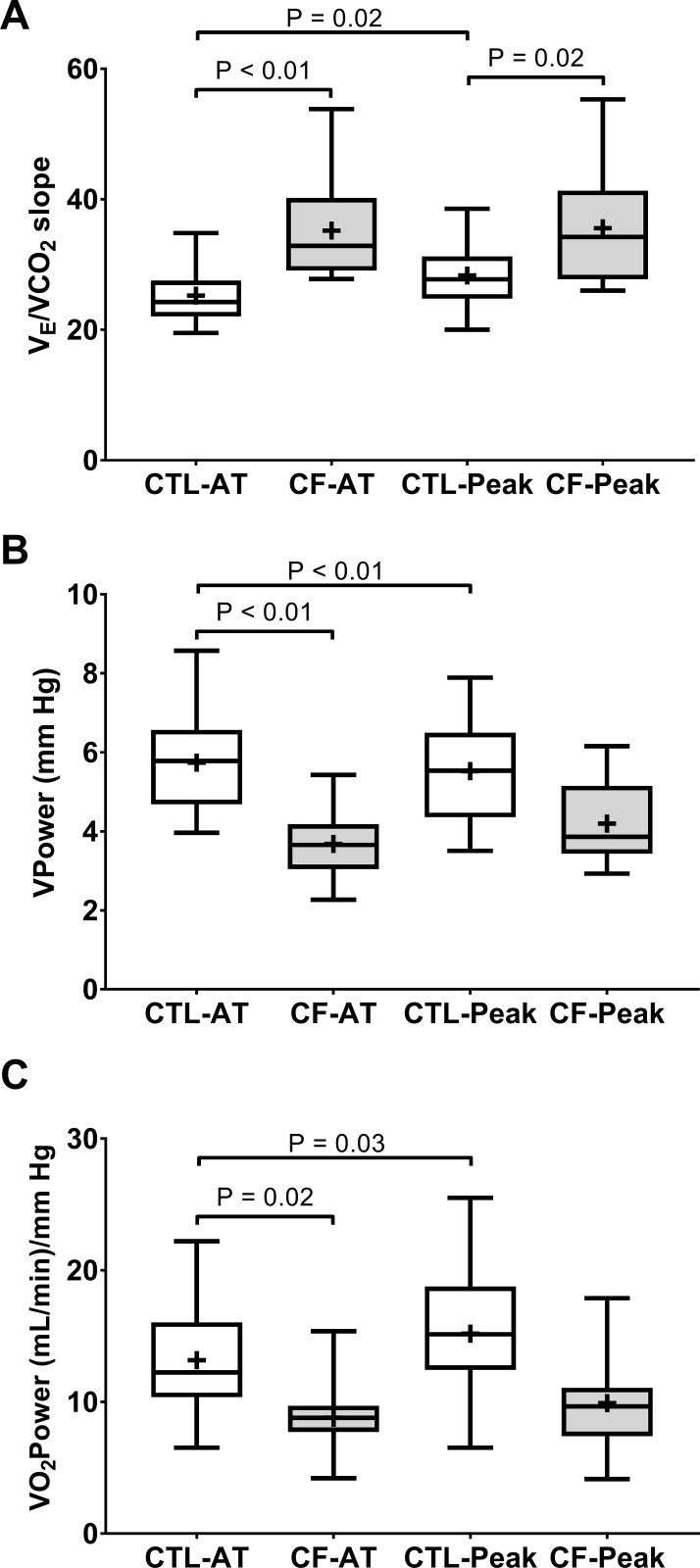
Advanced measures of cardiopulmonary function during CPET. Raw data are interquartile range with the center line representing the median and the cross symbol representing the sample mean. **A)** Slope of the ventilatory equivalent to carbon dioxide output ratio (${\dot{V}}_{E}/\dot{V}CO_{2}$ slope) from rest to either anaerobic threshold (AT) or peak exercise. **B)** Ventilatory power (VPower) at either AT or peak exercise. **C)** Peak oxygen uptake power ($\dot{V}O_{2}Power$) at AT or peak exercise. Healthy controls (CTL, N = 23); Cystic Fibrosis (CF, N = 16).

Illustrated in Fig 1A, the group-by-time interaction for ${\dot{V}}_{E}/\dot{V}CO_{2}$ slope was significant (*F =* 13.3, *P<*0.01), whereas Work_TOT_ (*F =* 2.0, *P* = 0.16) was not a significant covariate. This resulted in increased ${\dot{V}}_{E}/\dot{V}CO_{2}$ slope in CF compared to controls when assessed up to both AT and peak exercise; whereas ${\dot{V}}_{E}/\dot{V}CO_{2}$ slope did not differ between AT and peak exercise within CF, but did so within controls (Fig 1A).

In contrast, the group-by-time interaction (*F =* 13.7, *P<*0.01) with Work_TOT_ (*F =* 15.3, *P<*0.01) were significant for VPower (Fig 1B). Although this resulted in lower VPower in CF compared to controls at AT, this difference did not persist to peak exercise (Fig 1B). Additionally, consistent with higher ${\dot{V}}_{E}/\dot{V}CO_{2}$ slope comparing peak exercise to AT within controls in Fig 1A, VPower was reduced at peak exercise compared to AT within controls in Fig 1B. No within group differences were observed for VPower in CF.

Lastly, similar to VPower in Fig 1B, the group-by-time interaction (*F =* 8.0, *P<*0.01) with Work_TOT_ (*F =* 52.8, *P<*0.01) were significant for $\dot{V}O_{2}Power$ (Fig 1C). However, as illustrated in Fig 1C, increased $\dot{V}O_{2}Power$ at AT in controls compared to CF was because of higher $\dot{V}O_{2}$ (Table 2), not because of lower SBP (Table 3). This magnitude of increase from rest to AT for $\dot{V}O_{2}$ relative to SBP apparently did not persist to peak exercise, because despite $\dot{V}O_{2}$ remaining significantly higher at peak exercise in controls compared to CF, $\dot{V}O_{2}Power$ at peak exercise did not differ between controls and CF in Fig 1C.

**Table 3:** Rest, anaerobic threshold, and peak exercise heart rate and blood pressure responses in controls and Cystic Fibrosis.

	Rest			Anaerobic Threshold			Peak Exercise		
	Controls	CF	ES	Controls	CF	ES	Controls	CF	ES
HR, beats/min	82±3	92±7	0.60	147±10^‡^	125±7^‡^	1.18	180±5^†^^#^	154±5*^†^^#^	1.75
HR, % predicted peak	43±3	48±4	0.50	78±5^‡^	65±4^‡^*	1.28	95±3^†^^#^	80±4*^†^^#^	1.96
SBP, mm Hg	109±4	107±5	0.10	141±9^‡^	125±9^‡^	0.88	152±6^†^	144±13^†^	0.38
DBP, mm Hg	71±2	69±4	0.06	65±7	70±6	0.33	66±6	68±7	0.13
MAP, mm Hg	84±2	82±4	0.01	91±6^‡^	88±5	0.17	95±5^†^	93±7^†^	0.08
PulseP, mm Hg	38±3	38±4	0.15	76±11	55±8	0.96	86±10^†^	76±11^†^	0.41

Raw data are presented as mean ± 95% CL. Controls (n = 23); CF, cystic fibrosis (n = 16). ES, effect size; HR, heart rate; SBP, systolic blood pressure; DBP, diastolic blood pressure; MAP, mean arterial pressure; PulseP, pulse pressure.

* *P<*0.05, controls vs. CF.

^‡ ^*P<*0.05, rest vs. AT within group.

^† ^*P<*0.05, rest vs. peak exercise within group.

^# ^*P<*0.05, AT vs. peak exercise within group.

Significance after Tukey-Kramer post-hoc testing where appropriate.

#### Ventilation, gas-exchange, and oxygen saturation

Group-by-time interactions for $\dot{V}CO_{2}$ (*F =* 31.8, *P<*0.01), $\dot{V}CO_{2}$ (indexed to kg, *F =* 27.2, *P<*0.01), RER (*F =* 21.7, *P<*0.01), ${\dot{V}}_{E}$ (*F =* 39.3, *P<*0.01), ${\dot{V}}_{E}$ (indexed to kg, *F =* 34.5, *P<*0.01), ${\dot{V}}_{Ereserve}$ (*F =* 21.9, *P<*0.01), RR (*F =* 11.5, *P<*0.01), V_T_ (*F =* 16.8, *P<*0.01), V_T_ (indexed to kg, *F =* 17.6, *P<*0.01), P_ET_CO_2_ (*F =* 5.6, *P<*0.01), PECO_2_ (*F =* 7.3, *P<*0.01), PECO_2_/P_ET_CO_2_ (*F =* 4.7, *P<*0.01), and SpO_2_ (*F =* 6.1, *P<*0.01) were significant (Table 2). For all models, Work_TOT_ was a significant covariate except in models for RER (*F =* 1.8, *P =* 0.19), RR (*F =* 0.03, *P =* 0.87), PECO_2_/P_ET_CO_2_ (*F =* 1.4, *P =* 0.24), and SpO_2_ (*F =* 0.0, *P =* 0.98). Accordingly, after accounting for Work_TOT_ as a covariate in models, there were no between group differences for those parameters at peak exercise (Table 2).

#### Heart rate and blood pressure

Each group-by-time interaction for HR (*F =* 50.5, *P<*0.01), percent predicted peak HR (*F =* 56.4, *P<*0.01), SBP (*F =* 12.1, *P<*0.01), MAP (*F =* 5.4, *P<*0.01), and PulseP (*F =* 7.7, *P<*0.01) were significant, whereas DBP was not significant (*F =* 1.8, *P =* 0.14) (Table 3). In contrast, Work_TOT_ was a significant covariate in each of those models (*P<*0.01), except for MAP (*F =* 3.6, *P* = 0.07). This resulted in higher peak HR and percent predicted peak HR in controls compared to CF, whereas pairwise peak blood pressure differences were not significant (Table 3).

### Correlations between peak blood pressure and ventilation or gas-exchange

**Fig 2:**
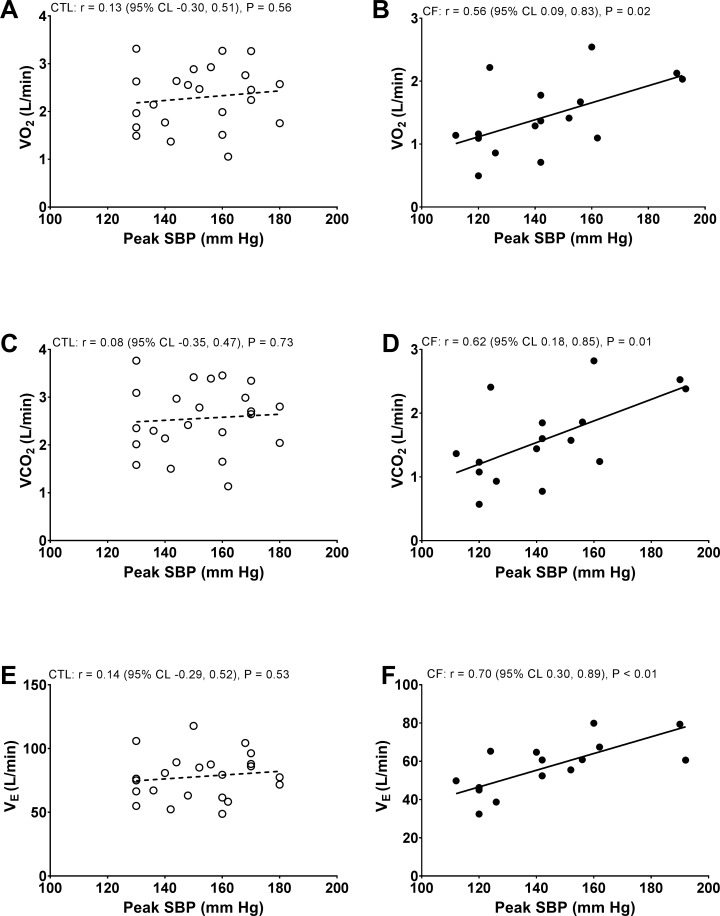
Pearson’s product moment correlation models. Between peak exercise systolic blood pressure (SBP) and peak exercise oxygen uptake ($\dot{V}O_{2}$), carbon dioxide output ($\dot{V}CO_{2}$), or minute ventilation (${\dot{V}}_{E}$) in healthy controls (CTL) or Cystic Fibrosis (CF).

For correlation models illustrated in Fig 2, there were no relationships between peak exercise SBP and peak $\dot{V}O_{2}$ (L/min), $\dot{V}CO_{2}$ (L/min), or ${\dot{V}}_{E}$ (L/min) in controls. Peak exercise SBP also did not correlate with other basic ventilatory or gas-exchange indices in controls ($\dot{V}O_{2}$ [mL/kg/min], *r* = -0.21; V_T_, *r* = 0.16; RR, *r* = 0.04; P_ET_CO_2_, *r* = 0.01; or PECO_2_, *r* = -0.06). In contrast, there were significant relationships between peak exercise SBP with peak $\dot{V}O_{2}$ (L/min), $\dot{V}CO_{2}$ (L/min), and ${\dot{V}}_{E}$ (L/min) in CF (Fig 2). Additional correlations between peak exercise SBP with peak $\dot{V}O_{2}$ (mL/kg/min) (*r* = 0.30), V_T_ (L) (*r* = 0.51), RR (*r* = 0.20), P_ET_CO_2_ (*r* = 0.41), or PECO_2_ (*r* = 0.31) were modest- to- moderate in CF.

**Fig 3:**
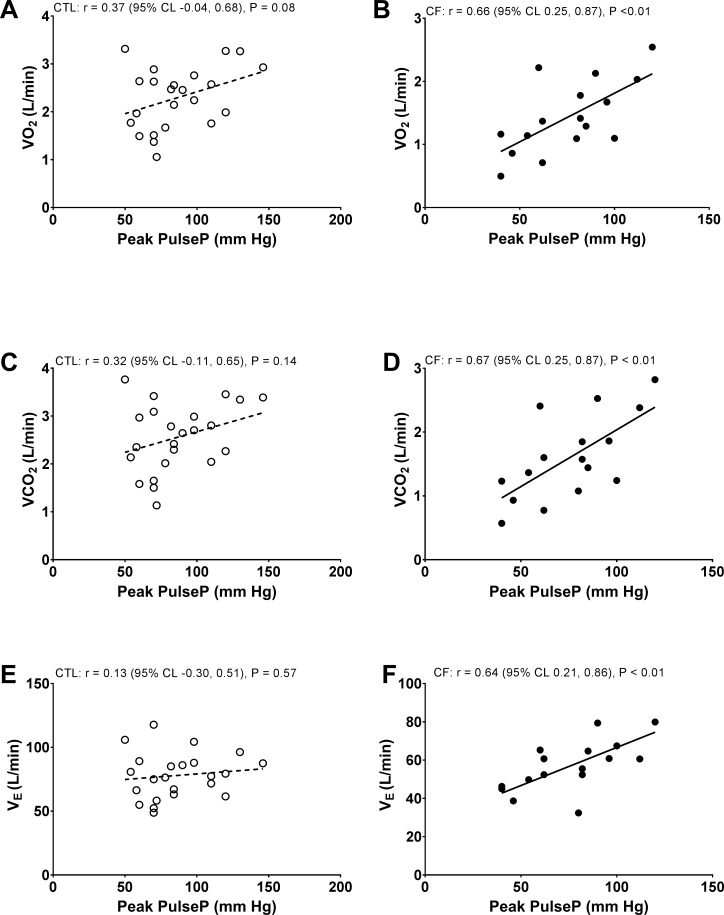
Pearson’s product moment correlation models. Between peak exercise pulse pressure (PulseP) and peak exercise oxygen uptake ($\dot{V}O_{2}$), carbon dioxide output ($\dot{V}CO_{2}$), or minute ventilation (${\dot{V}}_{E}$) in healthy controls (CTL) or Cystic Fibrosis (CF).

Consistent with peak exercise SBP correlations in Fig 2, peak exercise PulseP did not correlate with peak $\dot{V}O_{2}$ (L/min), $\dot{V}CO_{2}$ (L/min), or ${\dot{V}}_{E}$ (L/min) in controls (Fig 3). Peak exercise PulseP also did not correlate with peak $\dot{V}O_{2}$ (mL/kg/min) (*r* = 0.13); V_T_ (L) (*r* = 0.22), RR, (*r* = -0.05), P_ET_CO_2_, (*r* = 0.38), or PECO_2_ (*r* = 0.37) in controls. In contrast, peak exercise PulseP correlated with peak $\dot{V}O_{2}$ (L/min), $\dot{V}CO_{2}$ (L/min), or ${\dot{V}}_{E}$ (L/min) in CF in Fig 3. Whereas correlations between peak exercise PulseP with peak $\dot{V}O_{2}$ (mL/kg/min) (*r* = 0.64); V_T_ (L) (*r* = 0.46), RR (*r* = 0.15), P_ET_CO_2_ (*r* = 0.44), or PECO_2_ (*r* = 0.45) were modest- to- moderate in CF. Finally, peak exercise DBP did not demonstrate significant correlations in models for controls or CF.

## Discussion

A primary symptom in individuals with CF is low aerobic exercise capacity, which is related to quality of life and long-term prognosis in this population [8–14]. Although CF is commonly recognized as a genetic disease manifesting within the pulmonary system and primarily affecting pulmonary function, an accumulating body of evidence suggests the traditional paradigm linking reduced pulmonary function to decreased aerobic exercise capacity should also now consider integrated non-pulmonary factors as important contributors to this prognostic indicator in CF [8, 10–14, 18–25]. With this, observations from this study are consistent with the hypothesis that CF demonstrate a disease phenotype that extends beyond the lungs to cardiac, peripheral vascular, and skeletal muscle organ systems [8, 12–14, 18–25].

These data suggest three novel findings in young adults with mild- to- moderate severity CF. First, individuals with CF demonstrate a high ${\dot{V}}_{E}/\dot{V}CO_{2}$ slope at levels consistent with suggesting poor clinical status in adult patients with severe cardiopulmonary disease (e.g., ${\dot{V}}_{E}/\dot{V}CO_{2}$ slope ≥34 in HF or COPD) [26, 27, 29]. Second, despite CF demonstrating an augmented ${\dot{V}}_{E}/\dot{V}CO_{2}$ slope from rest to peak exercise, we did not observe reduced peak exercise VPower in CF compared to controls, suggesting a leftward shift in the peak SBP to Work_TOT_ relationship in CF. Lastly, at moderate- to- large magnitudes, peak exercise SBP and PulseP relate to peak exercise ventilation and gas-exchange in CF, providing further evidence to suggest there is calibration between central and peripheral mechanisms of exercise capacity in these individuals.

A consistent finding in studies across CF is the presence of abnormal resting airway function that may be explained by the genetic origins of this disease that cause deranged or absent CFTR within lung tissue [3–7]. Because of this well-known CF clinical phenotype, it has been traditionally assumed that amongst cardiovascular or pulmonary function indices irrespective of rest or exercise assessment that decreased resting FEV_1_, superseding all others, is the strongest predictor of CF clinical status (e.g., hospitalizations, exercise capacity, mortality, etc.) [5–7, 58–60]. However, despite this traditional understanding of CF, a clear physiologic translation from observations of low resting airway function to identification of pathophysiology provoking exacerbated exercise ventilation accompanied by decreased $\dot{V}O_{2peak}$ has not been keenly established in CF.

In several important respects, we support, and also extend the observations of others suggesting impaired resting airway function and depressed lung volumes are present in CF [5–7, 58–60]. However, and perhaps equally important, while others have illustrated that ${\dot{V}}_{E}$ with $\dot{V}CO_{2}$ as a ratio or slope during CPET may be increased in adolescent or adult CF [12–14, 23, 50], we demonstrate for the first time that increased blood pressure during CPET is directly related to elevated ventilation and, hence, appearing as an important contributor to augmented ${\dot{V}}_{E}/\dot{V}CO_{2}$ slope in adult CF. As such, consistent with observations and hypotheses of others [8, 12–14, 18–24], these data suggest compared to $\dot{V}O_{2peak}$, understanding the mechanisms of low ventilatory efficiency (e.g., high ${\dot{V}}_{E}/\dot{V}CO_{2}$ slope) may be equally important in elucidating the origins poor exercise capacity in CF. With this, new and noteworthy, in a complementary manner that extends emerging work in this field questioning peripheral vascular function in CF [19–21, 24, 36], these data suggest unresolved pathways involved in peripheral blood pressure function may serve an important role in contributing to abnormal ventilatory and gas-exchange responses to exercise in CF.

### Peripheral hemodynamic function at rest in Cystic Fibrosis

Although we observed abnormal blood pressure during exercise in CF, the precise mechanisms underlying the hypothesis of peripheral vascular dysfunction in CF remain largely unknown. Nevertheless, observations from Poore et al. [24] suggest that not only do adolescents with mild- to- moderate severity CF demonstrate attenuated resting FEV_1_, FEV_1_/FVC, and FEF_25-75_, linked to their decreased airway function, these individuals also demonstrate reduced brachial-artery flow-mediated dilation, suggesting the presence of vascular endothelial dysfunction. Consistent with observations of Poore et al. [24], pharmacologic studies in young adults with mild- to- moderate severity CF suggest these individuals have blunted reductions in systemic vascular resistance following acute inhalation of the β_2_-selective agonist albuterol, whilst implicating abnormal β_2_-adrenergic receptor function in failing to mediate vasodilatory control in response to acute changes in vasomotor tone [19]. Lastly, in an integrative study of experimental approaches akin to techniques of Poore et al. [24] and Van Iterson et al. [19], Rodriguez-Miguelez et al. [21] demonstrated in young adults with mild- to- moderate severity CF that relative changes in postocclusive reactive hyperemia, local thermal hyperemia, and acetylcholine iontophoresis of the resting arm are blunted in CF, suggesting the presence of impaired microvascular function in these individuals. Thus, while the exact physiologic and biomolecular pathways involved in explaining those observations warrant future study [19, 21, 24], because techniques such as flow-mediated dilation or administration of albuterol or acetylcholine represent targetable pathways of studying peripheral vascular function in CF, those data broadly suggest there may be peripheral vascular dysfunction related to attenuated vasodilatory reserve and impaired sympatholysis associated with CF.

### Peripheral hemodynamic function during exercise in Cystic Fibrosis

These data are initially in contrast to those of Hull et al. [20] and Schrage et al. [35], who in separate studies using cycle ergometry and handgrip exercise, respectively, illustrate blood pressure during exercise is similar in adults with mild- to- moderate severity CF compared to age matched controls. Young adults with CF trended at higher MAP during all forearm workloads compared to controls in Schrage et al. [35]; whereas, in Hull et al. [20], young adults with CF performed cycle ergometry at a significantly lower total workload compared to controls, yet blood pressure in either direction did not differ between groups. Thus, particularly in the study of Hull et al. [20], it is possible that workload adjusted blood pressure in CF may have resulted in between group differences resembling or possibly exceeding increased blood pressure in the present study. Equally intriguing, Hull et al. [20] also demonstrated that CF tended to have higher large arterial stiffness (i.e., augmentation index) compared to controls during exercise, which could reasonably contribute to high systemic vascular resistance and elevated blood pressure in this population. Nevertheless, while those data of Hull et al. [20] are encouraging in suggesting alternative origins of abnormal peripheral vascular function and blood pressure control in CF, use of arterial waveforms in non-invasive modeling of arterial stiffness or other hemodynamic parameters (e.g., central/peripheral blood pressures) during dynamic upright leg ergometry has not been clearly validated or replicated in individuals with CF.

Augmented blood pressure during exercise as a result of impaired vasodilation, arterial stiffness, and/or exaggerated sympathetically-mediated vasoconstriction is complex with the potential of leading to, or being the consequence of numerous effects at the local skeletal muscle level in CF [12, 18, 22, 23, 25]. For example, impaired sympatholysis may lead to decreased perfusion and convective delivery of oxygen to metabolically active skeletal tissue, resulting in and/or contributing to attenuated oxygen diffusion across the capillaries into skeletal muscle [61, 62]. In this manner, although exploratory and potentially hypothesis generating based on these and other data in CF [12, 18, 22, 23, 25], reduced oxygen availability at aerobically active tissue could lead to exaggerated recruitment of anaerobic pathways for energy generation resulting in increased production and circulation of CO_2_ and accumulation of harmful metabolic byproducts (e.g., increased hydrogen ion) [62, 63]. Thus, when increased arterial CO_2_ is accompanied by blunted VO_2_ and/or gas transfer at the alveoli level, this could potentiate neural-mediated (e.g., central/peripheral chemoreflex) disproportionate elevations in ventilation and, hence, a high ${\dot{V}}_{E}/\dot{V}CO_{2}$ slope [62, 63].

### Limitations

Individuals with CF homozygous for the ΔF508 genotype comprised 75% of our sample, which is one of 1000+ possible genotypes associated with this disease [1, 2]. Therefore, it remains unclear how genotype may be related to the outcomes of this study. Nevertheless, it is estimated that the ΔF508 CFTR genotype comprises approximately 70% of CF diagnoses [1, 2], making our CF sample similar to estimates of the general CF population. Nevertheless, we acknowledge that the findings of this study remain generalizable to this sample of adults with mild- to- moderate severity CF, and that further studies inclusive of individuals with CF across the age and disease severity spectrum are warranted to establish the clinical implications of these data across the CF population.

In this context, it is also important for future research to elucidate not only a mechanistic understanding of what underlies a high ${\dot{V}}_{E}/\dot{V}CO_{2}$ slope specifically in CF, but it is also necessary to establish standardized methods and thresholds demarcating what could be interpreted as an exaggerated ${\dot{V}}_{E}/\dot{V}CO_{2}$ slope response that are sensitive to differences in age and disease severity of study samples. This is critical because while we demonstrate augmented ${\dot{V}}_{E}/\dot{V}CO_{2}$ slope when calculated using all data up to either the AT or peak exercise in adult CF, others either have [50] (from rest to peak exercise, but not to AT or lower) or have not [64] (neither from rest to AT or to peak exercise) observed ${\dot{V}}_{E}/\dot{V}CO_{2}$ slope to be increased in adolescent CF. As such, while there is no clear body of evidence to refute our suggestion that ${\dot{V}}_{E}/\dot{V}CO_{2}$ slope responses in this study were abnormally elevated in adult CF, we acknowledge that we interpreted ${\dot{V}}_{E}/\dot{V}CO_{2}$ slope values using recognized thresholds established in other cardiopulmonary disease patient populations to support our conclusions [26–29].

Despite the validity of PECO_2_ and the PECO_2_/P_ET_CO_2_ ratio to estimate ventilation and perfusion matching and/or physiologic deadspace to V_T_ ratio as demonstrated by Hansen et al. [55], we did not invasively assess any potential adjustments in ventilation and perfusion matching or directly sample blood gases, which could be used to confirm our relationships between blood pressure and ventilation and gas-exchange. Although our PECO_2_/P_ET_CO_2_ ratios at peak exercise did not suggest a remarkable presence of ventilation and perfusion mismatch due to high physiologic deadspace to V_T_ ratios, if marked ventilation and perfusion mismatch is present in CF, the ability to directly quantify the direction of this shift would add tremendous value in being able to better objectively describe contributions from pulmonary abnormalities during CPET in these individuals. As such, this is an important next direction for this line of study as it cannot be assumed that any presence of ventilation and perfusion mismatch may be a sole consequence of ventilatory limitations without considering the potential role of reduced cardiac function. An accumulating body of work in this field provides evidence that CF may demonstrate abnormal cardiac function secondary to pulmonary limitations [14, 19, 25, 33, 34]. Thus, elucidating the integrative role that peripheral hemodynamic function has on abnormal cardiac and pulmonary responses during CPET is warranted in CF. Lastly, specific studies focusing on vascular tissue stiffness [20], endothelial function [24], peripheral capillary membrane diffusing capacity to understand peripheral oxygen kinetics, and *in vivo* mitochondrial function (e.g., oxidative phosphorylation capacity) are needed to better phenotype the periphery in CF.

## Conclusions

Young adults with mild- to- moderate severity CF demonstrate exaggerated ${\dot{V}}_{E}/\dot{V}CO_{2}$ slope and blunted $\dot{V}O_{2}$ during CPET. New and noteworthy, correlating with this rise in ${\dot{V}}_{E}$ and coupled to ${\dot{V}}_{E}/\dot{V}CO_{2}$ slope, these data further suggest CF demonstrate augmented peripheral blood pressure (i.e., SBP, MAP, and PulseP) during CPET. Although the underlying pathophysiologic mechanisms cannot be elucidated from this study, these data provide novel insights potentially linking abnormal peripheral blood pressure function to abnormal ventilation and gas-exchange patterns during exercise in CF. Additional large scale studies in CF across the age and disease severity spectrum are needed to confirm the influence of this disease on the development of comorbidities associated with increased blood pressure such as hypertension and cardiovascular disease.
